# Testing the predictive power of reverse screening to infer drug targets, with the help of machine learning

**DOI:** 10.1038/s42004-024-01179-2

**Published:** 2024-05-09

**Authors:** Antoine Daina, Vincent Zoete

**Affiliations:** 1 Molecular Modeling Group, SIB Swiss Institute of Bioinformatics, CH-1015 Lausanne, Switzerland; 2 Computer-Aided Molecular Engineering, Department of Oncology UNIL-CHUV, Ludwig Institute for Cancer Research Lausanne Branch, University of Lausanne, Lausanne, Switzerland

**Keywords:** Target identification, Cheminformatics, Cheminformatics, Computational chemistry

## Abstract

Estimating protein targets of compounds based on the *similarity principle*—similar molecules are likely to show comparable bioactivity—is a long-standing strategy in drug research. Having previously quantified this principle, we present here a large-scale evaluation of its predictive power for inferring macromolecular targets by reverse screening an unprecedented vast external test set of more than 300,000 active small molecules against another bioactivity set of more than 500,000 compounds. We show that machine-learning can predict the correct targets, with the highest probability among 2069 proteins, for more than 51% of the external molecules. The strong enrichment thus obtained demonstrates its usefulness in supporting phenotypic screens, polypharmacology, or repurposing. Moreover, we quantified the impact of the bioactivity knowledge available for proteins in terms of number and diversity of actives. Finally, we advise that developers of such approaches follow an application-oriented benchmarking strategy and use large, high-quality, non-overlapping datasets as provided here.

## Abstract (web summary)

Ligand-based reverse screening plays an important role in predicting molecular targets of bioactive compounds, however, its predictive ability might be overlooked due to the limitations of existing external test sets. Here, the authors assess the predictive power of reverse screening with a large diverse external bioactivity dataset.

## Introduction

The importance of predicting primary and secondary macromolecular targets of therapeutic compounds was clearly demonstrated by retrospective analyses defining the number of known protein targets for drugs^1,2^. Underlying concepts, such as polypharmacology, specificity or repurposing, are considered throughout any modern drug R&D project. This also concerns the initial stages of discovery where the number of molecules to evaluate is massive, but the physical samples are scarce, prompting the use of fast yet robust bioinformatic models.

Whereas earlier studies about target and bioactivity prediction were conducted^3–6^, the game-changing work of Shoichet and colleagues^7–10^ on ligand-based reverse screening was accompanied by a remarkable experimental effort to confirm about half of the predicted off-target effects of 656 drugs among 73 possible proteins. Since then, a plethora of computational tools followed by expert opinions were released^11–13^. This research area is no exception to the growing *penchant* for unsupervised methods calling for due warnings about black-box and overfitting pitfalls. The scientific output has focused on meticulously comparing machine-learning algorithms with sophisticated stratification of the bioactivity knowledge^1,14–18^. The actual predictive ability has been strikingly overlooked, probably due to the difficulty of constructing appropriate external test sets.

To address this methodological shortcoming, we propose the first assessment of the predictive power of ligand-based reverse screening for the estimation of small molecule drug targets with a vast, diverse, curated, external bioactivity dataset.

## Results and discussion

### Training

The target prediction engine evaluated here is a logistic model combining shape and chemical similarity^19^ and trained on data curated from the ChEMBL database^20^. This method relies on the *Similarity Principle*, which was quantified by us previously for several molecular descriptors (including ES5D vectors and FP2 fingerprints, see below)^21^. The robustness of this machine-learning model was scrupulously confirmed by cross-validation several times^19^ and was recognized by peers as one of the most carefully statistically validated methods in the field^15^.

Here, ChEMBL was mined to obtain the training data comprising 501,959 compounds showing experimental bioactivity against 3669 protein targets. (see *Methods, Data extraction*, Supplementary Fig. 1a).

For each of the 501,959 compounds, the tridimensional shape and projection of physicochemical properties were translated into twenty 18-dimension float vectors following the ElectroShape approach (ES5D vectors)^22^. As well, the chemical structure of each compound was encoded as one 1024-bit binary vector (FP2 fingerprints) (Supplementary Fig. 1b)^23^. Pair-wise comparisons between all compounds produced the 3D-Score matrix with Manhattan-based similarity values of ES5D vectors (for the closest of 20 conformations), and the 2D-Score matrix with Tanimoto coefficients of FP2 fingerprints (see *Methods, Chemoinformatics*, Supplementary Fig. 1c).

To address the variation in contributions of these descriptors in the regression with molecular size^19^, 51 subsets were created, each corresponding to a given number of heavy atoms in the first (“query”) molecule of every pair (see *Methods, training methodology*). For each subset, a binary logistic model was trained to find the best constant *C* and coefficients (*c*_*1*_, *c*_*2*_) for the regression features (*3D-Score* and *2D-Score*) (Supplementary Fig. 1d). To reduce noise from training, the final coefficients for calculating the probability of predictions were obtained by fitting the *C*, *c*_*1*_ and *c*_*2*_ curves via a third-degree polynomial function (Supplementary Fig. 2a).

The high internal classification ability measured by 10-fold cross-validated Matthews correlation coefficient for each 51 size-related subset (MCC_cv_, see *Methods, training methodology*, Supplementary Fig. 2b, Supplementary Table 1) confirmed the robustness of the approach as defined several time^19,24,25^. The lower MCC, precision and recall for the lesser heavy atom classes have already been observed and related to the poorer protein specificity of very small ligands^26^ and partly to less populated classes (Supplementary Fig. 2c).

### External validation

The output of the regression model is an unbiased computed probability. Accordingly, we established a strategy to assess the predictive ability that reflects the applicative scope, i.e. reverse screening to predict the most probable protein targets for as many active compounds as possible. The rank of experimental targets in a list of predicted proteins ordered by calculated probability was recorded. Noteworthy, for this reverse screening evaluation exercise, the calculated probability values are only used as a scoring scale to rank the predicted targets.

The mining of Reaxys^®^ enabled the construction of an unprecedentedly vast test set^27^. Applying filters comparable to those used for the training set, we retrieved high-quality data for 364,201 small molecules, not included in the ChEMBL training set, yet active on 1180 human proteins shared with ChEMBL (See *Methods, Data extraction*).

**Fig. 1:**
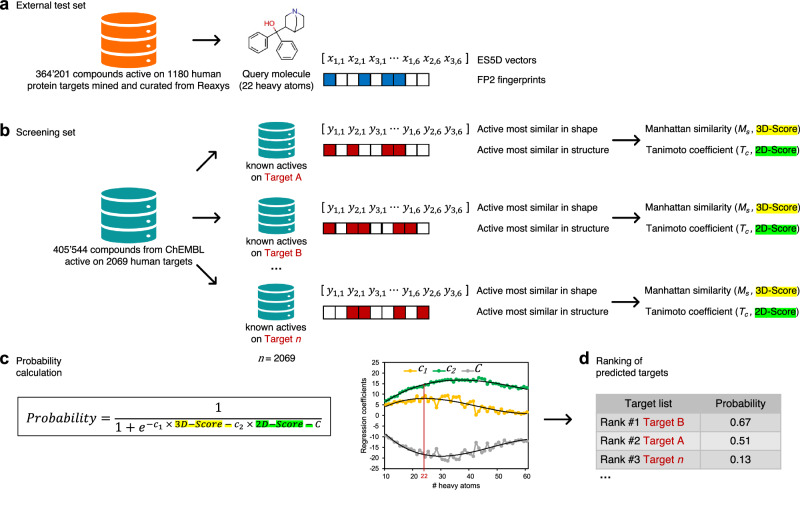
Predictive ability evaluation method on large external test set. **a** Bioactivity data extraction from Reaxys (version 03.2019) applying filtering criteria: molecules between 5 and 80 heavy atoms, active at 10 µM or less as IC_50_, EC_50_, K_i_, K_D_, K_ic_ or K_m_ in a binding assay on a well-defined protein or protein complex shared with the ChEMBL 25 training set. 364,201 unique compounds active on 1180 human protein targets retrieved as 2-dimensional SDF submitted to removal of counter ions or solvents, kekulization and neutralization to calculate path-based binary molecular fingerprints up to 7 atoms (FP2 fingerprints). 20 all-atom conformers generation to calculate 20 shape vectors of 18 dimensions (${x}_{n,p}$), with ${x}_{n,p}$ the average distance to the order *n* between all atoms and the *p*^th^ of six centroids (ES5D vectors). **b** Reverse-screening of each Reaxys test compound against the ChEMBL screening set organized by known actives per target, in order to find the actives most similar in 3D and in 2D for each of the 2069 screened targets (can be the same or different molecules). **c** The highest computed Manhattan similarity value and Tanimoto coefficient are inputted in the logistic equation as *3D-Score* and *2D-Score* features, respectively. The probability of a given protein to be targeted by the “query” compound is calculated using with the final *c*oefficients (*c*_*1*_, *c*_*2*_ and *C*) obtained by training and curve-smoothing as a function of the number of heavy atoms in the query molecule (Supplementary Fig. 1 and Supplementary Fig. 2). **d** The actual output for assessing the predictive ability (Fig. 3) is the list of the 2069 screened proteins ranked from the highest to the lowest calculated probability for each test compound for which the experimental target(s) are known.

This external test set was reverse-screened against the fraction of the ChEMBL set active on human proteins (*i.e*. the screening set, see *Methods, Testing strategy*). The ES5D vectors and FP2 fingerprints of each 364,201 test compound (Fig. 1a) were compared to all 405,544 compounds of the screening set to find the most similar known actives on every 2069 ChEMBL human targets in terms of shape and chemical structure (Fig. 1b). For each protein target, the highest Manhattan-based similarity value and Tanimoto coefficient were inputted in the logistic equation as *3D-Score* and *2D-Score* features, respectively (Fig. 1c). By using the coefficients (*c*_*1*_, *c*_*2*_ and *C*) corresponding to the number of heavy atoms in the test compound, the probability was calculated for all 2069 proteins to rank them from most probable to least probable targets (Fig. 1d).

### Physicochemical and chemical spaces

To ensure that the applicability domain of the model is respected, and that the validation exercise matches with the “real-life” application—finding probable targets for medicinal-chemistry-oriented bioactive small molecular compounds—the respective physicochemical spaces covered by the training set and the test set were compared.

The distributions of seven molecular and physicochemical descriptors for both sets are depicted in Supplementary Fig. 3, as a function of the number of molecules, and as per percentage of total set (see *Physicochemical description* in the *Methods* section). These indicate the clear overlap of the two molecular sets in every descriptor dimension. More precisely and as shown in Table 1, the distributions of lipophilicity, saturation, flexibility, apparent polarity, hydrogen-bonding capacity and size are very similar between the training set and the test set. This is quantified by very negative Z-factors^28^ for *n*-octanol/water partition coefficient (WLOGP), the fraction of sp3 carbon (fCsp3), the number of rotatable bonds, the polar surface area (TPSA), and the number of hydrogen-bond acceptors (HBA) and donors (HBD), as well as for molecular weight (MW). This confirms that the test set falls in the applicability domain of the predictive model with very comparable physicochemical spaces covered by both extensive molecular sets.

**Table 1:** Distribution of physicochemical properties among training and test molecules

Descriptor	Training set (*n* = 501,959)		Test set (*n* = 364,201)		Z-factor
	Average	Std dev	Average	Std dev	
WLOGP	4.16	2.029	3.74	1.933	−27.36
fCsp3	0.33	0.185	0.34	0.170	−70.99
# rot. bonds	7.08	4.280	7.66	3.978	−41.60
TPSA [A^2^]	93.83	46.259	97.72	41.506	−66.68
HBA	5.30	2.408	5.81	2.352	−26.98
HBD	1.82	1.568	1.81	1.448	−603.40
MW [g/mol]	430.91	118.385	465.72	107.227	−18.45

Beside physicochemical space, the chemical diversity between the two sets was measured by two different types of molecular scaffolds (see *Scaffold computation* in the *Methods* section). According to the Murcko wire-like frameworks^29^, the 501,959 training molecules are described by 25,046 scaffolds, and the 364,201 test molecules by 21,820 scaffolds. As per the more abstract Oprea approach^30^, the training molecules are described by 38,896 scaffolds, and the test molecules by 33,754 scaffolds. Relatively and for both definitions, the test set is more chemically diverse with an average of 16.7 and 10.8 molecules per Murcko and Oprea scaffolds, respectively, compared to 20.0 and 12.9 molecules per Murcko and Oprea scaffolds for the training set.

Moreover, the common scaffolds between both sets are 10,317 Murcko scaffolds (41.2% of the training set and 47.3% of the test set, Supplementary Fig. 4a) and 15,004 Oprea scaffolds (38.6% of the training set and 44.5% for the test set, Supplementary Fig. 4b). With less than half of both sets overlapping according to two different molecular scaffold definitions, the training and the test sets can be considered as chemically distinct from each other.

**Fig. 2:**
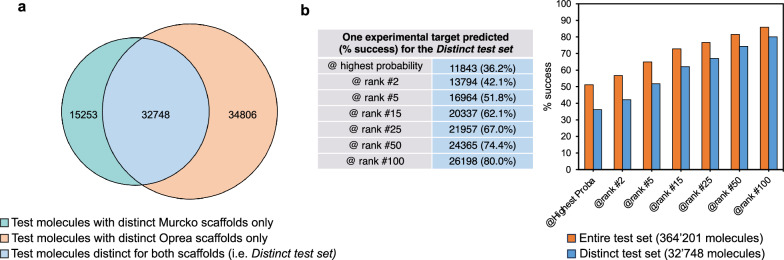
The *Distinct test set* and its impact on reverse screening success rate. **a** Number of compounds in the test set described by distinct scaffolds not extracted from any training molecule. The *Distinct test set* contains 32,748 molecules (9% of the entire test set) described by Murcko^29^ and Oprea^30^ scaffolds not represented in the training set. **b** Success in predicting one experimentally validated target for all 32,748 distinct test compounds compared to the entire test set (values in Fig. 3a).

Furthermore, by considering the scaffolds from the test set that do not describe any training compound, 11,503 unique distinct Murcko scaffolds can be extracted from 48,001 test molecules (13.2%), and 18,750 unique distinct Oprea scaffolds from 67,554 test molecules (18.5%). Finally, 32,748 test molecules (termed as the *Distinct test set* and representing 9.0% of the entire test set, see Fig. 2a) can be considered strictly chemically distinct from the training set, since they are described by Murcko and Oprea scaffolds, which do not describe any training compound. This indicates further the relevance of building a vast external test set from a distinct source. Even when applying strict criteria for molecular diversity (unrelated to the model itself, as here, two orthogonal definitions of scaffolds), the predictive ability assessment can be also performed on numerous external compounds objectively defined as chemically distinct from the training data (see section *Predictive ability*).

Noteworthy, the bioactivity data at the root of the external test set were severely constrained in many dimensions. The most drastic reductions took place in the bioactivity spectrum and the chemical space. The former regards the selection of targets shared with the training set only (1180 among the more than 14,000 human proteins targeted by at least one small molecular compound available in Reaxys version 03.2019) and the latter was the selection of external actives not part of the training set (364,201 compounds among the almost 7 million small molecules with bioactivity data on a well-defined target). Despite these radical reduction measures, the external test set remains large as well as chemically diverse and distinct from the training molecules.

These chemical and physicochemical examinations demonstrate the relevance of the evaluation exercise, core of this study, with an external test set that is not only large, chemically diverse and distinct from the training set but also that falls in the applicability domain of the logistic model according to seven molecular and physicochemical properties. The predictive ability assessment strategy proposed reproduces the real objective of the reverse screening methodology, i.e. finding the probable protein targets of bioactive small molecular compounds, in the context of drug discovery and medicinal chemistry.

### Predictive ability

**Fig. 3:**
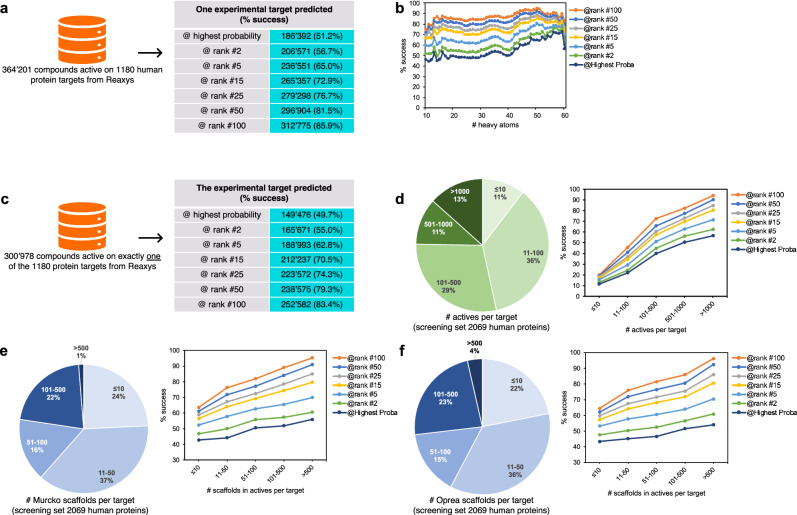
Global predictive ability of ligand-based reverse screening. **a** Success in predicting one experimentally validated target—among all known targets—for all 364,201 bioactive compounds external to the training/screening sets, as a function of the position in the list of proteins ranked by probability calculated via the logistic model (Fig. 1). **b** Percentage of success at different ranks as a function of the molecular size (Supplementary Fig. 1d) showing the relationship between the number of heavy atoms in the query molecule and the predictive ability. **c** Success in predicting the target for the 300,978 compounds from the external test set having exactly one experimental target. **d** Amount of bioactivity knowledge available on targets. Distribution of the 2069 screened targets with respect to their number of active compounds in ChEMBL, and the impact on the predictive ability on the Reaxys active test compounds, showing much higher success rate for targets with numerous known ligands. **e** Extent of chemical diversity among ligands of targets. Distribution of the 2069 screened targets with respect to the number of Murcko scaffolds^27^ among their active compounds in ChEMBL, and the impact on the predictive ability on the Reaxys active compounds showing higher success rate for targets with actives displaying numerous molecular scaffolds. **f** Same analysis on chemical diversity conducted with Oprea scaffolds^28^ leading to the same conclusion that higher predictive ability is obtained for protein target with chemically diverse actives displaying numerous molecular scaffolds.

The global predictive ability can be quantified by the success in retrieving, by reverse-screening, one of the experimental targets of bioactive query molecules among the predicted most probable ones. Remarkably, for 51.2% of the test compounds, the predicted protein with the highest probability was indeed a validated target (Fig. 3a). This predictive capacity is considerably higher than the 0.1% expected from a random ranking. The success becomes 72.9% within the predicted 15 most probable proteins, as typically displayed in Web interfaces^24,25^. The success rate versus rank plateaus, reaching 85.9% at rank #100 (Supplementary Fig. 5).

The predictive ability is essentially constant along the classes of heavy atoms in the test compounds (Fig. 3b), with a noisier signal for smaller molecules and a slight increase for larger molecules. The most stable signal is obtained for molecules containing between 20 and 40 heavy atoms, which are the most populated classes (see Supplementary Fig. 2c and Supplementary Fig. 6 for training set and test set, respectively) and those corresponding to drugs and druglike molecules^31^.

The relationship between the performance of ligand-based reverse screening and the chemical novelty of submitted compounds has long been published^19^. However, the size, diversity, and chemical nature of the here-built test set justify a renewed evaluation with many more test molecules objectively chemically distinct from the training set. According to the chemical diversity study described in section *Physicochemical and chemical spaces*, 9% of the entire test set involves compounds described by molecular scaffolds that cannot be extracted from any molecule in the training set, with respect to both the Murcko and the Oprea definitions (Fig. 2a). The large size of the whole test set made it possible to build a so-called “distinct test set” containing as many as 32,748 molecules. Predicting protein targets of these compounds, chemically strictly unrelated to the training set (nor the screening set, which is a subset of the training set), increases a priori the difficulty of the exercise. Expectedly as shown in Fig. 2b, the success is less for the “distinct test set” than for the entire test set. Nevertheless, the success rate is still very acceptable with a correct target predicted for more than one third (36.2%) of the distinct molecules at highest probability, for more than half of the distinct molecules (51.8%) at rank #5, for more than two-third of the distinct molecules (67.0%) at rank #15, finally reaching 80.0% at rank #100. Gratifyingly, this further confirms the robustness of the prediction generated by the method, even outside of the chemical space covered by the training data. This demonstrates the usefulness and relevance of target prediction by ligand-based reverse-screening for new compounds close to the state-of-the-art in me-too projects, but also for novel chemotypes in more prospective drug discovery.

Considering only test compounds with exactly one experimental target reported in Reaxys broadens the scope of analysis while retaining a vast external test set (300,978 compounds) (Fig. 3c). Retrieving the correct single target is a more difficult exercise than finding one among several, however the predictive ability remains very high with a success rate of 49.7% at highest probability. Moreover 70.5% and 83.4% of success at rank #15 and rank #100 correspond to a list of estimated protein targets enriched by 55 and 10 folds, respectively. This level of enrichment demonstrates the practicality of reverse virtual screening to provide useful guidance and focus on relevant proteins in experiments like the deconvolution of phenotypic screens, the setup of polypharmacology panels, or the selection of repurposing targets (remarkable applicative examples^32,33^).

A central question for any ligand-based approach is how the predictive performance varies with the amount of knowledge available. The capacity of reverse screening to predict the correct target improves dramatically as the pool of known ligand expands (Fig. 3d). For targets having 11 to 100 known active compounds (36% of the proteins in the screening set), the success is 40.0% at highest probability and 72.5% at rank #100 (an 8.5-fold enrichment). The success is much higher when the proteins to predict have a lot of bioactivity data available like for those with more than a thousand actives (13% of the proteins in the screening set) with 56.3% at highest ranking and 93.9% at rank #100 (an 11-fold enrichment). Conversely, proteins with only few known ligands, like 10 or less actives (11% in the screening set) show substantially lower success rates with 11.4% at highest ranking and 19.8% at rank #100 (a 2.3-fold enrichment). This is a measure of the difficulty to find a very similar active molecule by screening on targets for which only few are known.

The chemical diversity of actives for a given target was also investigated through two distinct molecular scaffolds, the Murcko wire-like frameworks^29^ and the more abstract Oprea approach^30^ (Fig. 3e and f). Both analyses lead to the same conclusion that the more diverse the actives, the higher the predictive ability. In both analyses, at rank #15, one can expect a success of about 65% if the target has between 11 to 50 scaffolds among its actives ( ~ 36% of the proteins). The success rate drops to about 55% when the number of scaffolds is 10 or less, but increases up to about 80% when the number of scaffolds is larger than 500 (only 1–4% in the ChEMBL screening set).

For the first time, the relationship between the performance of ligand-based target prediction and the size and diversity of the bioactivity space is quantified. This is a strong incentive to populate specialized databases with bioactive chemicals and targets as diverse as possible, rather than focusing only on the number of molecules or proteins. Noteworthy though, the probability calculated through the logistic equation is not affected per se by the number of experimentally active compounds. It is enough to find one active molecule by reverse screening to calculate a prediction, whose relevance relies on the molecular similarity. However, in applicative target prediction tools, it is common practice to impose a limit of similarity below which a known active of the screening is considered dissimilar and does not enter the calculation of probability, mainly for reducing the time of computation^25^.

The extent of bioactivity knowledge useful to be reverse screened must be balanced with data quality. The demonstrated excellent capacity to predict targets of bioactive molecules is bound to improve further with continued efforts made on the quantity, the diversity and the quality inside specialized resources. For experts, we strongly suggest extracting open-access data from e.g. ChEMBL^20^ or PubChem^34^, or broadly distributed data like in Reaxys, applying strict filtering criteria (see *Methods*, *Data extraction*).

Besides having validated and quantified the predictive ability of ligand-based reverse-screening methods at large scale for the first time, we recommend that developers of machine-learning target prediction approaches follow the application-oriented validation strategy (see *Methods, Testing strategy*) and use large, high-quality, diverse and non-overlapping bioactivity datasets (e.g. both provided datasets from ChEMBL and Reaxys, used here for training and testing, respectively) for future development, validation, and benchmarking studies.

The results and material provided here call for consolidating this bioinformatic method as a valid and mature machine-learning approach in drug research but also in the many applications in biology and chemistry where the protein targets of small molecules require to be estimated. Finally, this supervised machine-learning technology has proven simple and fast enough for implementation behind websites. Simple Web interfaces, like the pioneer Similar Ensemble Approach (SEA, https://sea.bkslab.org)^8^ or the extensively used and referenced SwissTargetPrediction (http://www.swisstargetprediction.ch)^24,25^ can quickly provide trustful predictions for routine work or for non-experts in the field. Of note, The SwissTargetPrediction webtool has been cited 2260 times (according to Clarivate®, accessed March 19, 2024); 94% were research articles, 78% of them in the categories “Pharmacology, Medicinal Chemistry, Chemistry (Multidisciplinary), Biochemistry and Molecular Biology” suggesting experimental studies including validation of the computational predictions, like in refs. ^32,33^. Publications in other categories comprise reviews of medical experts explaining the use SwissTargetPrediction for drug repurposing in their branch, for instance in cardiology^35^ or engineers supporting the choice and underlying the performance of SwissTargetPrediction’s unique logistic model among other ML algorithms in their *Experimentalist’s Guide to Machine Learning for Small Molecule Design*^36^.

## Methods

### Data extraction

The ChEMBL database^20^ version 25 was chosen as the data source for training the machine-learning and for screening library, for three main reasons: (i) the open-sourceness enables unrestricted availability for anyone; (ii) various previous versions of the predictive engine evaluated in this work have been built on ChEMBL data, some of which are in the backend of the renown and much used SwissTargetPrediction webtool^24,25^; (iii) the content is contemporaneous with the granted access to the Reaxys database (version 03.2019), which has been an opportunity to shape an unprecedented large external set from high-quality data from a different source yet of similar origin, i.e. medicinal chemistry-related bioactivity knowledge.

ChEMBL and Reaxys raw content is differently annotated and organized, however it has been possible to homogenize them by applying filtering criteria. We used MySQL requests to extract bioactivity data from a local copy of ChEMBL 25 for compounds with 5 to 80 heavy atoms tested in a binding assay (tagged “B” and confidence score >3) on a human, rat or mouse macromolecular target (single protein or protein complex). Bioactivity information for 501,959 unique small molecule compounds was thus extracted: 452,656 actives with IC_50_, EC_50_, K_i_ or K_D_ ≤ 10 µM, and 46,165 considered inactives with IC_50_, EC_50_, K_i_ or K_D_ ≥ 100 µM. In between is a “gray area” of 3138 compounds that were considered neither active nor inactive. Comparable filters were applied to Reaxys 03.2019. Only active compounds were retrieved with IC_50_, EC_50_, K_i_, K_D_, K_ic_ or K_m_ ≤ 10 µM, tagged with type *binding*, *enzymatic*, *generic*, *second messenger*, *electrophysiology* or *transactivation*, and category in vitro. In accordance with the validation objective of this work, molecules present in the ChEMBL set were removed from the Reaxys set using the Obgrep program (OpenBabel version 2.4.1)^23^ and the JChem Search utility (version 21.3, www.chemaxon.com). Moreover, only data points involving targets shared with the ChEMBL training set were retained for the test set. This was achieved by human curation and mapping on UniProt identifiers^37^. Information about 364,201 compounds active on 1180 human proteins was thus gathered. Of note, the massive reduction of the Reaxys data in response to the need of this study resulted into using only 5% of the compound having bioactivity data recorded in version 03.2019, and 8% of the human proteins targeted by bioactive small molecules as included in version 03.2019. Importantly, all 1180 targets of the Reaxys test set are *findable* since part of the ChEMBL screening data whereas each 364,201 test compound was confirmed external to the training and screening sets (see *Chemoinformatics* section).

### Chemoinformatics

The molecular information included in the bioactivity data extracted as detailed above were submitted to further standardization treatments, identical for both sources. The isomeric SMILES obtained from ChEMBL and the two-dimensional SDF from Reaxys were unsalted, desolvated, neutralized, kekulized with JChem Microservices Structure manipulation tools (version 21.3, www.chemaxon.com) and stored as two separate flat files including all extracted values together with IDs. The training set includes all information from ChEMBL describing the bioactivity of 501,959 unique compounds on 3669 proteins. The test set includes the SMILES, ReaxysID, the number of targets and their UniProt identifiers derived from Reaxys content for 364,201 compounds active on 1180 proteins.

To describe the chemical structure of the compounds, each standardized SMILES were then transformed as molecular fingerprints by the path-based FP2 method implemented in OpenBabel (version 2.4.1), which encodes the presence or absence of linear fragments from 1 to 7 atoms^23^. These FP2 fingerprints were stored as individual 1024-bit binary strings (Fig.1a and Supplementary Fig. 1b).

Using JChem Microservices Structure manipulation and Chemical calculations tools (version 21.3, www.chemaxon.com), each standardized SMILES was then protonated as at pH 7.4 before generating the 20 lowest energy conformations, which were stored as multi-MOL2 files. To describe the shape and the spatial projection of physicochemical properties, every conformer of each compound was encoded into a float vector according to the ElectroShape 5D procedure^22^ as detailed several times^19,24,25^. In brief, distances are computed between each atom and six centroids encompassing the structure in a 5-dimentional space (three Cartesian coordinates, as well as atomic charge^38^ and lipophilic contribution^39^). The average, the standard deviation and the third moment of all distances for one conformation are stored in an ES5D vector of 18 dimensions (${x}_{n,p}$), where ${x}_{n,p}$ is the average distance to the order *n* between all atoms and the *p*^th^ centroid (Fig.1a and Supplementary Fig. 1b).

Noteworthy, the completeness of the extraction and standardization procedure was verified *a posteriori* by analyzing the pairs of molecules where the Tanimoto coefficient (${T}_{c}$) on FP2 fingerprints equals to 1.000. These cases were either: i) one molecule is a large substructure of the other (a known limitation of path-based fingerprints); or ii) the compounds are different salts or solvation forms of the same parent molecule; or iii) the compounds differ by stereochemistry. In all cases, both compounds have been kept since not linked to the same bioactivity as for both ChEMBL and Reaxys entries. The same molecule was never found.

### Physicochemical description

The physicochemical spaces covered by the training set and the test set were measured by seven descriptors (See Supplementary Fig. 3). The SwissADME web tool^40^ was used to calculate the molecular weight (MW), the *n-*octanol/water partition coefficient (WLOGP)^39^, the topological polar surface area (TPSA)^41^, the number of rotatable bonds, the fraction of *sp*^*3*^ carbon (fCsp3), the number of H-bond acceptors (HBA) and the number of H-bond donors (HBD), for the 501,959 training compounds and the 364,201 test compounds.

The overlap of each descriptor distributions between the training set and the test set was quantified by Z-factor^28^, calculated according to Eq. (1), (see Table 1).

1 $$Z{\mbox{-}}{factor}=1-\frac{3({\delta }_{{tr}}+{\delta }_{{ts}})}{\left|{\mu }_{{tr}}-{\mu }_{{ts}}\right|}$$

where, *σ*_tr_ is the standard deviation of the descriptor values in the training set; *σ*_ts_ is the standard deviation of the descriptor values in the test set; *µ*_tr_ is the average of the descriptor values in the training set; *µ*_ts_ is the average of the descriptor values in the test set.

### Training methodology

Two similarity matrices were computed by pair-wise comparisons between all 501,959 compounds of the ChEMBL training set described by shape (ES5D vectors) and chemical structure (FP2 fingerprints) (Supplementary Fig. 1c). For shape comparison, the 3D-Score similarity matrix is built with the highest Manhattan-based similarity values (${M}_{{s}_{i,j}}=1/(1+\frac{1}{18}{d}_{i,j})$), where ${d}_{i,j}$ is the smallest Manhattan distance between all 20 × 20 pairs of ES5D vectors, each encoding a different conformation for molecules *i* and *j*. For chemical structure comparison, the 2D-Score matrix contains the Tanimoto coefficients (${T}_{{c}_{i,j}}$) between all FP2 fingerprints of pairs for molecules *i* and *j*.

The ChEMBL training set was split into subsets, each one corresponding to a given number of heavy atoms in the first (“query”) molecule of every pair. Subsets were thus prepared from 11 to 59 heavy atoms; smaller molecules were grouped in one class ( ≤ 10 heavy atoms) and larger molecules in another one ( ≥ 60 heavy atoms) to finally define 51 size-dependant training subsets (Supplementary Fig. 1d). Each compound in the training subset (active or inactive) was compared to all known actives of each target. Inactive compounds were defined as having an experimental activity higher than or equal to 100 µM (see *Methods*, *Data extraction*), or as not being reported active by ChEMBL in any binding assay on the protein under consideration (i.e. alleged inactives). The ratio of 10 inactives for 1 active—previously defined empirically as most suited^19^—was applied. For every comparison, ${M}_{{s}_{i,j}}$ and ${T}_{{c}_{i,j}}$ were retrieved, and the highest values for each similarity metric considered as the features of the model i.e. *3D-Score* and *2D-Score*, respectively. Practically, each line of a subset training file regards one training compound and one target, and reports the *3D-Score*, *2D-Score*, and “1” or “0” to indicate whether this query molecule is active or inactive on that target.

For each of the 51 subsets, a binary logistic model was trained to find the best regression constant *C* and coefficients (*c*_*1*_, *c*_*2*_) for both features (*3D-Score* and *2D-Score*), according to the Eq. (2), where *Probability* of being active on a given target is “1” or “0” for all training compounds. The default parameters of the *LogisticRegression* function of the scikit-learn program (version 0.23.2) were used.

2 $${Probability}=\frac{1}{1+{e}^{-{c}_{1}\times 3D-{Score}-{c}_{2}\times 2D-{Score}-C}}$$

The internal robustness was monitored by 10-fold cross-validation. Matthews correlation coefficients (MCC) were calculated with Eq. (3) and were averaged over the 10 cross-validation sets (MCC_cv_, Supplementary Fig. 2b). As well averaged precision and recall were calculated with Eq. (4) and Eq. (5), respectively. The Supplementary Table 1 provides, for each size-related training subsets, the MCC_CV_ with standard deviation, together with precision and recall. It should be noted that the 10 folds are random, given the construction of the subsets, which are shuffled and contain no information about molecule or target.

3 $${MCC}=\frac{{TA}\times {TI}-{FA}\times {FI}}{\sqrt{({TA}+{FA})\times ({TA}+{FI})\times ({TI}+{FA})\times ({TI}+{FI})}}$$

4 $${Precision}=\frac{{TA}}{{TA}+{FA}}$$

5 $${Recall}=\frac{{TA}}{{TA}+{FI}}$$

where, *TA* is the number of known actives returning *Probability* > 0.5; *TI* is the number of inactives returning *Probability* ≤ 0.5; *FA* is the number of inactives returning *Probability* > 0.5; *FI* is the number of known actives returning *Probability* ≤ 0.5

To reduce the noise from the training, the final coefficients to be employed for calculating probability of predictions were obtained by fitting the *C*, *c*_*1*_ and *c*_*2*_ curves via a third-degree polynomial function. This way a set of final coefficients for predicting targets are obtained for each of the 51 subsets (Supplementary Fig. 2a).

### Testing strategy

All 364,201 active compounds of the Reaxys external test set were reverse screened towards the screening set, which corresponds to the active part of the ChEMBL training set organized by known actives per human target (in total 405,544 molecules active on 2069 proteins, Fig. 1b). For this, the 20 ES5D vectors which encode the shape of the query molecule were compared to the 20 ES5D vectors of all ChEMBL active compounds on one target. The highest ${M}_{s}$ corresponding to the active most similar in shape is considered as the *3D-Score* parameter. Similarly, the FP2 fingerprints describing the chemical structure of the query compound is compared to the FP2 fingerprints of all ChEMBL active compounds on one of the 2069 targets of interest. The highest ${T}_{c}$ value corresponds to the active most similar in structure and is considered as the *2D-Score* parameter. The probability for the protein to be targeted by the query molecule is obtained by inputting both parameters (*3D-Score* and *2D-Score*) in the logistic Eq. (2) together with the final coefficients and constant (c*1*, c*2* and *C*) obtained by training and curve-smoothing from the subset corresponding to the number of heavy atoms in the query molecule (Fig. 1c).

The search for the most similar actives according to the shape or to the chemical structure (which can be the same or two different compounds) and the calculation of the probability were repeated independently for all the 2069 protein targets of the screening set. The final output of this testing workflow is a list of the 2069 possible targets ranked from the most probable to the least probable. The quantified predictive ability of the ligand-based reverse screening is defined from the ranks of the known experimental targets for all 364,201 external test compounds (Fig. 3). It is important to note that in the context of this reverse screening, and for the core of the study presented here, the calculated probability values are considered as scores with the only objective to rank the different predicted targets.

### Scaffold computation

Two different scaffold definitions were applied to the molecules of both the training set and the test set. All Standardized SMILES (see *Chemoinformatics* section) were submitted to the strip-it program (version 1.0.2, www.silicos-it.be) to extract the wire-like frameworks as proposed by Bemis and Murcko^29^ (*MURCKO_2* definition), and the more abstract Oprea scaffolds^30^ (*OPREA_2* definition). This enabled the description of the chemical space covered by both molecular sets (Supplementary Fig. 3) and the creation of the so-called “*Distinct test set*” with 32,748 external test compounds described by distinct scaffolds not extracted from any training molecule, according to both the Murcko and the Oprea definitions (Fig. 2a). This subset (9% of the entire test set) allowed for the evaluation of the success rate of the ligand-based reverse screening for predicting targets of molecules objectively chemically different from the training set (Fig. 2b). As well, the predictive capacity of reverse screening with respect to the chemical diversity of actives for a given target was made possible by grouping the scaffolds of the known actives per proteins (Fig. 3e and Fig. 3f).

### Supplementary information

Supplementary Information

## Data Availability

Bioactivity data were obtained from the ChEMBL (version 25) and the Reaxys (version 03.2019) databases for training/screening and testing, respectively. A short extract of the raw ChEMBL data for training is given in Supplementary Table 2 to show three lines corresponding to an active, an inactive and a “gray area” datapoints, respectively. Processed data have been deposited in a Zenodo repository (10.5281/zenodo.7534175). The screening set file contains, for each active compound, the standardized SMILES, the ChEMBLID, the number of experimental target(s) and their UniProt identifier(s). Similarly, the test set file contains, for each active compound, the ReaxysID, the number of experimental target(s) and the UniProt identifier(s). For Reaxys users, the chemical structure can be obtained through bulk request on the corresponding website. Access to www.reaxys.com and to Reaxys data can be obtained by contacting Elsevier directly. The first 300 entries also display the standardized SMILES so that every reader can reproduce the results obtained by the reverse screening exercise. The construction of logistic models was performed on the data described here by strictly following the steps detailed in the methodological articles^19,24,25,42^ and their supplementary materials.

## References

[1] Peón A, Dang CC, Ballester PJ (2016). How reliable are ligand-centric methods for target fishing?. Front. Chem..

[2] Mestres J, Gregori-Puigjané E, Valverde S, Solé RV (2009). The topology of drug-target interaction networks: implicit dependence on drug properties and target families. Mol. Biosyst..

[3] Schuffenhauer A, Floersheim P, Acklin P, Jacoby E (2003). Similarity metrics for ligands reflecting the similarity of the target proteins. J. Chem. Inf. Comput. Sci..

[4] Horvath D, Jeandenans C (2003). Neighborhood behavior of in silico structural spaces with respect to in vitro activity spaces−a novel understanding of the molecular similarity principle in the context of multiple receptor binding profiles. J. Chem. Inf. Comput. Sci..

[5] Paolini GV, Shapland RHB, Hoorn WP, van, Mason JS, Hopkins AL (2006). Global mapping of pharmacological space. Nat. Biotechnol..

[6] Oprea TI, Tropsha A, Faulon J-L, Rintoul MD (2007). Systems chemical biology. Nat. Chem. Biol..

[7] Hert J, Keiser MJ, Irwin JJ, Oprea TI, Shoichet BK (2008). Quantifying the Relationships among Drug Classes. J. Chem. Inf. Model..

[8] Keiser MJ (2007). Relating protein pharmacology by ligand chemistry. Nat. Biotechnol..

[9] Keiser, M. J. et al. Predicting new molecular targets for known drugs. *Nature* **462**, 175–182 (2009). 10.1038/nature08506 PMC2784146 19881490

[10] Lounkine E (2012). Large-scale prediction and testing of drug activity on side-effect targets. Nature.

[11] Byrne R, Schneider G (2019). In silico target prediction for small molecules. Methods Mol. Biol..

[12] Comess KM (2018). Emerging approaches for the identification of protein targets of small molecules - a practitioners’ perspective. J. Med. Chem..

[13] Sydow D (2019). Advances and challenges in computational target prediction. J. Chem. Inf. Comput. Sci..

[14] Sturm N (2020). Industry-scale application and evaluation of deep learning for drug target prediction. J. Cheminform..

[15] Mathai N, Chen Y, Kirchmair J (2020). Validation strategies for target prediction methods. Brief. Bioinforma..

[16] Mervin LH, Afzal AM, Engkvist O, Bender A (2020). Comparison of scaling methods to obtain calibrated probabilities of activity for protein-ligand predictions. J. Chem. Inf. Comput. Sci..

[17] Ye, Q., Zhang, X. & Lin, X. *Intelligent Computing Theories and Application, 17th International Conference, ICIC 2021*, Lecture Notes in Computer Science, Vol. 12838, (eds Huang, D. S., Jo, K. H., Li, J., Gribova, V. & Premaratne, P.) 87–99 (Springer, Cham, 2021).

[18] Yang S-Q (2021). Current advances in ligand-based target prediction. WIREs Comput Mol. Sci..

[19] Gfeller D, Michielin O, Zoete V (2013). Shaping the interaction landscape of bioactive molecules. Bioinformatics.

[20] Mendez D (2019). ChEMBL: towards direct deposition of bioassay data. Nucleic Acids Res.

[21] Bragina ME, Daina A, Perez MAS, Michielin O, Zoete V (2022). The SwissSimilarity 2021 web tool: novel chemical libraries and additional methods for an enhanced ligand-based virtual screening experience. Int J. Mol. Sci..

[22] Armstrong MS, Finn PW, Morris GM, Richards WG (2011). Improving the accuracy of ultrafast ligand-based screening: incorporating lipophilicity into ElectroShape as an extra dimension. J. Comput Aided Mol. Des..

[23] O’Boyle NM (2011). OpenBabel: An open chemical toolbox. J. Cheminform..

[24] Gfeller D (2014). SwissTargetPrediction: a web server for target prediction of bioactive small molecules. Nucleic Acids Res..

[25] Daina A, Michielin O, Zoete V (2019). SwissTargetPrediction: updated data and new features for efficient prediction of protein targets of small molecules. Nucleic Acids Res..

[26] Nobeli I, Favia AD, Thornton JM (2009). Protein promiscuity and its implications for biotechnology. Nat. Biotechnol..

[27] *Reaxys*. (Copyright © 2023 Elsevier Limited except certain content provided by third parties. Reaxys® is a trademark of Elsevier Limited.).

[28] Zhang J-H, Chung TDY, Oldenburg KR (1999). A Simple Statistical Parameter for Use in Evaluation and Validation of High Throughput Screening Assays. SLAS Discov..

[29] Bemis GW, Murcko MA (1996). The properties of known drugs. 1. Molecular frameworks. J. Med. Chem..

[30] Pollock SN, Coutsias EA, Wester MJ, Oprea TI (2008). Scaffold topologies. 1. Exhaustive enumeration up to eight rings. J. Chem. Inf. Comput. Sci..

[31] Leeson PD (2021). Target-Based Evaluation of “Drug-Like” Properties and Ligand Efficiencies. J. Med Chem..

[32] Carotenuto P (2022). Targeting the MITF/APAF-1 axis as salvage therapy for MAPK inhibitors in resistant melanoma. Cell Rep..

[33] Bhunia D (2018). Spatial position regulates power of tryptophan: discovery of a major-groove-specific nuclear-localizing, cell-penetrating tetrapeptide. J. Am. Chem. Soc..

[34] Kim S (2020). PubChem in 2021: new data content and improved web interfaces. Nucleic Acids Res.

[35] Abdelsayed M, Kort EJ, Jovinge S, Mercola M (2022). Repurposing drugs to treat cardiovascular disease in the era of precision medicine. Nat. Rev. Cardiol..

[36] Lindley SE, Lu Y, Shukla D (2023). The experimentalist’s guide to machine learning for small molecule design. ACS Appl. Bio Mater..

[37] Consortium U (2019). UniProt: a worldwide hub of protein knowledge. Nucleic Acids Res.

[38] Halgren TA (1998). Merck molecular force field. I. Basis, form, scope, parameterization, and performance of MMFF94. J. Comput. Chem..

[39] Wildman SA, Crippen GM (1999). Prediction of physicochemical parameters by atomic contributions. J. Chem. Inf. Comput. Sci..

[40] Daina A, Michielin O, Zoete V (2017). SwissADME: a free web tool to evaluate pharmacokinetics, drug-likeness and medicinal chemistry friendliness of small molecules. Sci. Rep..

[41] Ertl P, Rohde B, Selzer P (2000). Fast calculation of molecular polar surface area as a sum of fragment-based contributions and its application to the prediction of drug transport properties. J. Med. Chem..

[42] Gfeller D, Zoete V (2015). Protein homology reveals new targets for bioactive small molecules. Bioinformatics.

