# Supplementary material for: Testing the predictive power of reverse screening to infer drug targets, with the help of machine learning
Source: Commun Chem. 2024 May 9;7:105. doi: 10.1038/s42004-024-01179-2 (PMC11082207; doi:10.1038/s42004-024-01179-2)
Supplement: Supplementary file 1 — Supplementary Information [file 42004_2024_1179_MOESM1_ESM.pdf]

Supplementary Information to :

Testing the predictive power of reverse screening to infer drug targets, with the help of machine learning

Antoine Daina[1], Vincent Zoete[1,2,*]
[1]Molecular Modeling Group, SIB Swiss Institute of Bioinformatics, CH-1015 Lausanne, Switzerland.
[2]Computer-Aided Molecular Engineering, Department of Oncology UNIL-CHUV, Ludwig Institute for Cancer Research Lausanne Branch, University of Lausanne, Switzerland
*Corresponding author. vincent.zoete@unil.ch

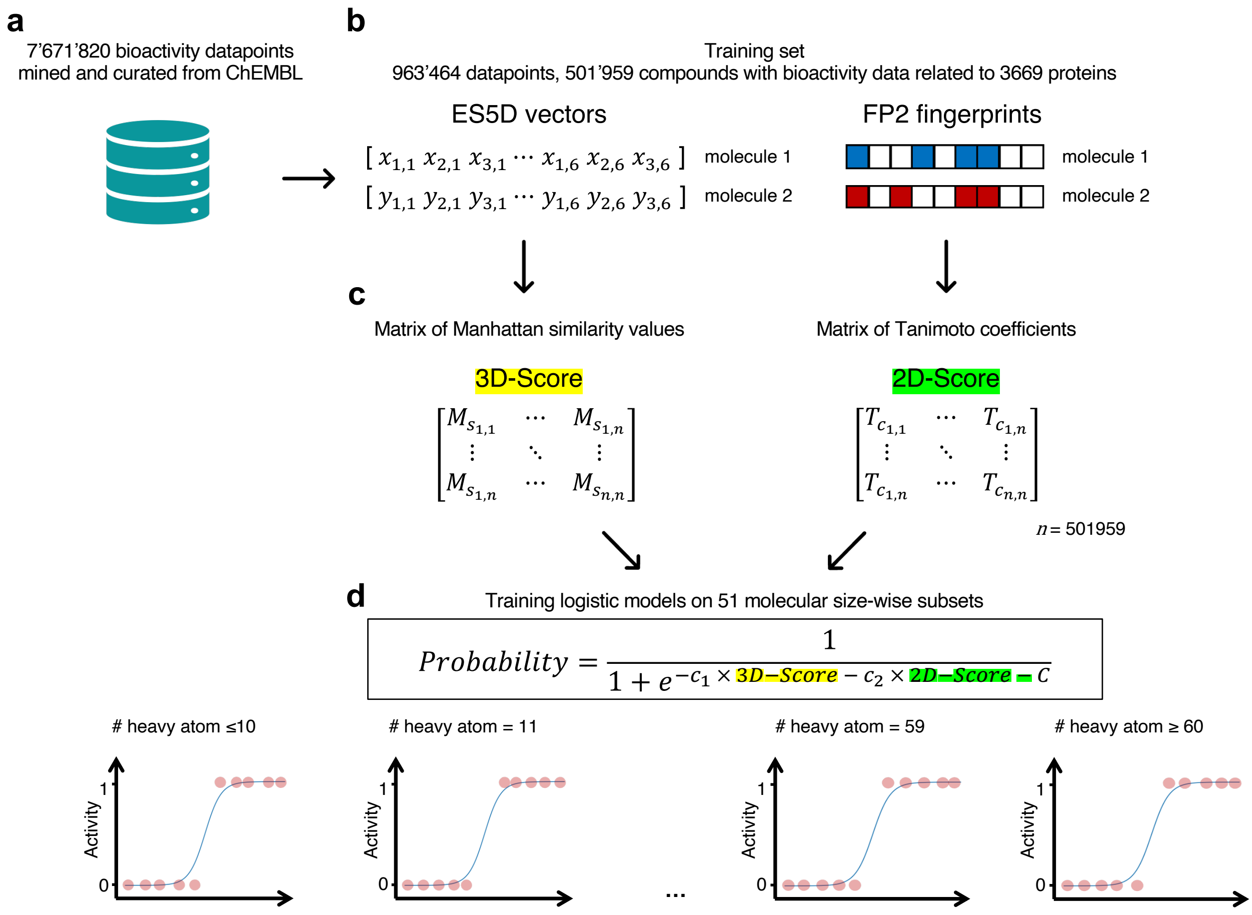

Supplementary Fig. 1: Training of logistic models. a, Bioactivity data extraction from ChEMBL 25 applying filtering criteria: molecules between 5 and 80 heavy atoms, with IC_{50}, EC_{50}, K_i or K_D value in a binding assay showing a confidence score >3, on a well-defined protein or protein complex. **b**, SMILES curation involving standardization, removal of counter ions or solvents, neutralization, kekulization to calculate path-based binary molecular fingerprints up to 7 atoms (FP2 fingerprints). 20 all-atom conformers generation to calculate 20 shape vectors of 18 dimensions ($x_{n,p}$) of the average distance to the order *n* between all atoms and the pth of six centroids (ES5D vectors). **c**, 3D-Score similarity matrix is constructed by calculating similarity values ($M_{s_{i,j}} = 1/(1 + \frac{1}{18} d_{i,j})$), where $d_{i,j}$ is the smallest Manhattan distance between all pairs of conformer vectors for molecules *i* and *j* (from 1 to *n*=501’959). 2D-Score similarity matrix is constructed by calculating Tanimoto coefficient ($T_{c_{i,j}}$) between all pairs *i* and *j* (from 1 to *n* = 501’959). **d**, Training of the logistic model within the 51 subsets related to the number of heavy atoms in the first molecule of every pair from which both features (*3D-Score* and *2D-Score*) were extracted. Active compounds (1) are those with recorded bioactivity value ≤ 10μM on a given target; inactive compounds (0) are those with recorded bioactivity value ≥ 100μM on a given target or those not reported by ChEMBL as active in any binding assay on the protein under consideration. The ratio of 10 inactives for 1 active was enforced.

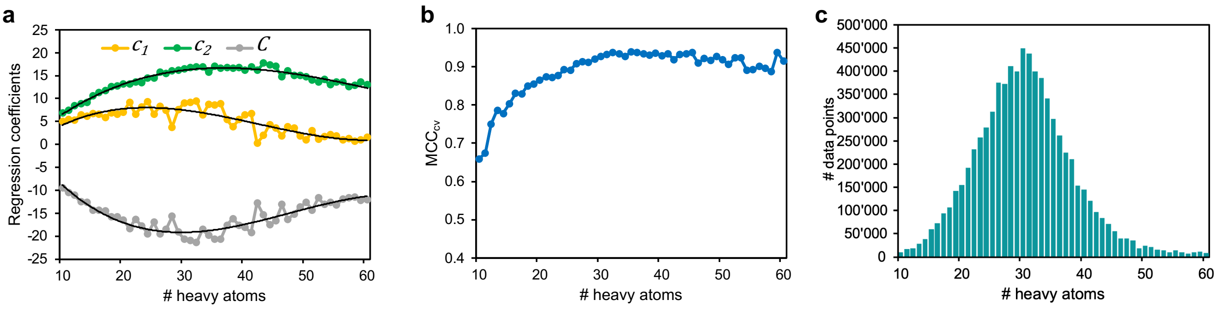

Supplementary Fig. 2: Logistic model and internal robustness. a, Dots represent raw regression coefficients for 3D-score (c_1) and 2D-Score (c_2) and constant (C) as a function of the number of heavy atoms in the "query" molecule obtained by the logistic equation for every 51 size-related training subset. Curves are smoothed through third-degree polynomial functions to generate the final coefficients for prediction (**Fig. 1c**). **b**, internal classification ability measured as Matthews correlation coefficients obtained through 10-fold cross-validation (MCC_{CV}) as a function of the number of heavy atoms in the "query" molecule. **c**, Total number of datapoints (one actives per ten inactives) in training subsets.

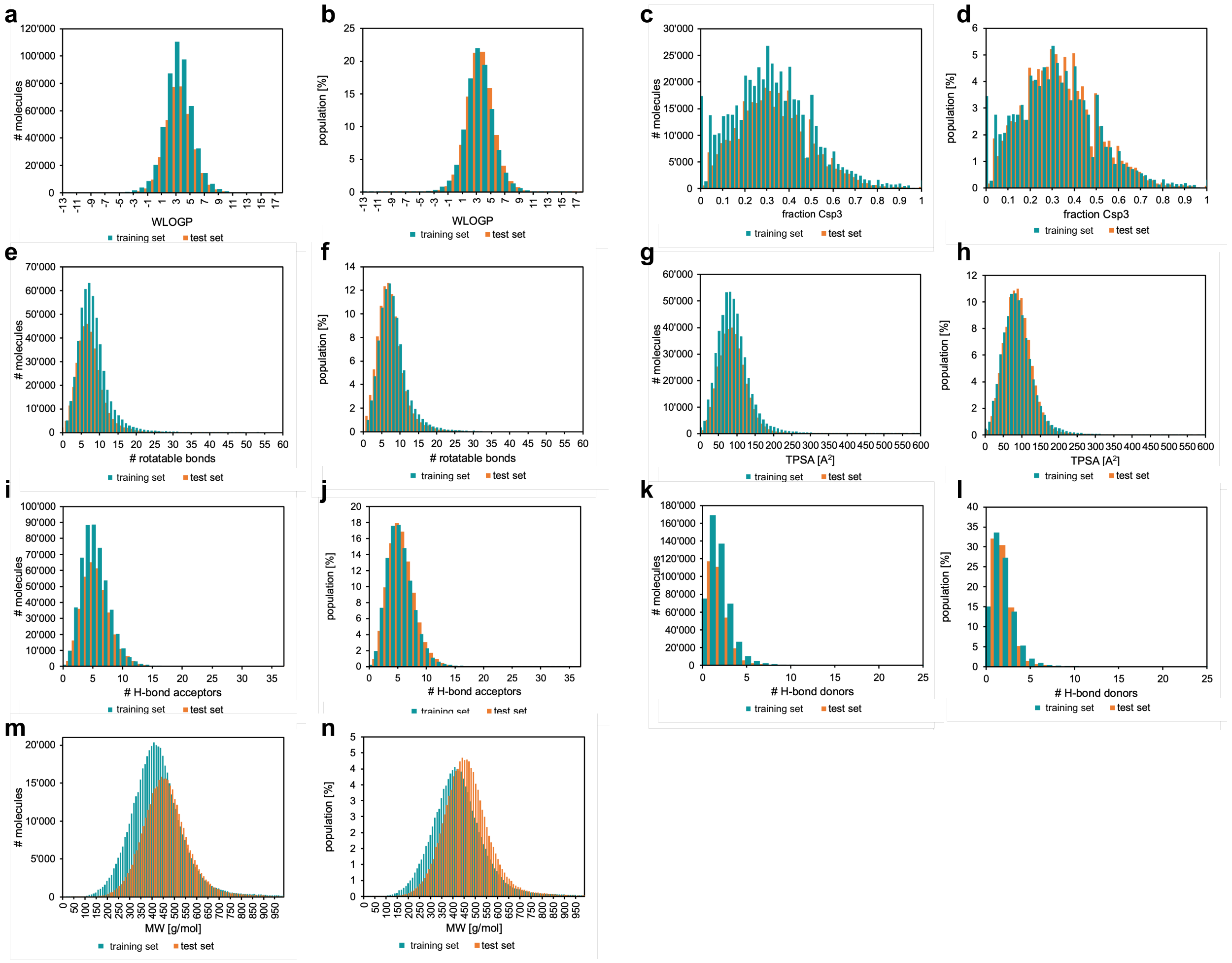

Supplementary Fig. 3: Physicochemical space of the training set and the test set. Absolute and relative distribution of seven descriptors for **a.** and **b.** lipophilicity (WLOGP), **c.** and **d.** saturation (fraction Csp3), **e.** and **f.** flexibility (# rotatable bonds), **g.** and **h.** apparent polarity (TPSA), **i.** and **j.** number of H-bond acceptors, **k.** and **l.** number of H-bond donors, **m.** and **n.** size (MW).

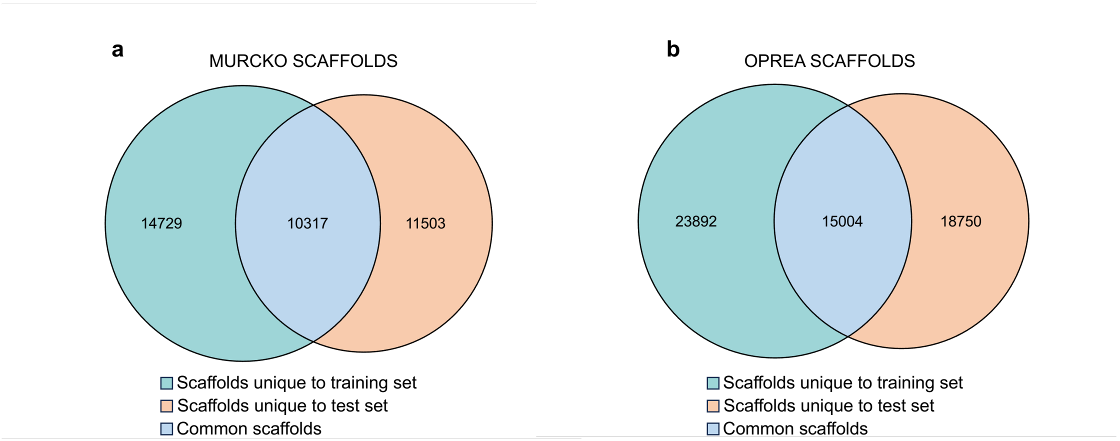

Supplementary Fig. 4: Chemical space of the training set and the test set. Repartition of scaffolds according to **a.** the Murcko wire-like frameworks, and **b.** the Oprea approach.

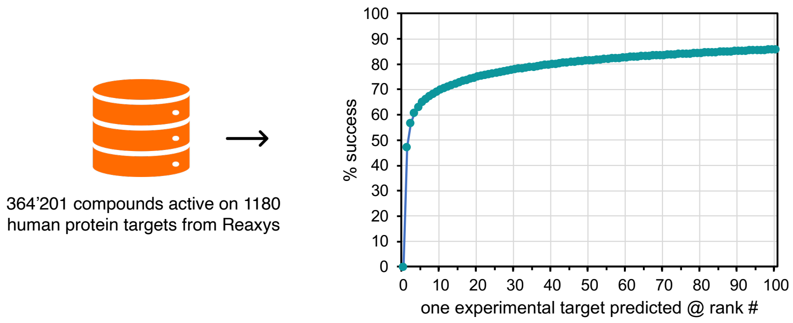

Supplementary Fig. 5: **Predictive ability** defined as the success in retrieving one experimental target, as recorded in Reaxys, within the n-best ranked targets according to probability calculated by the logistic regression.

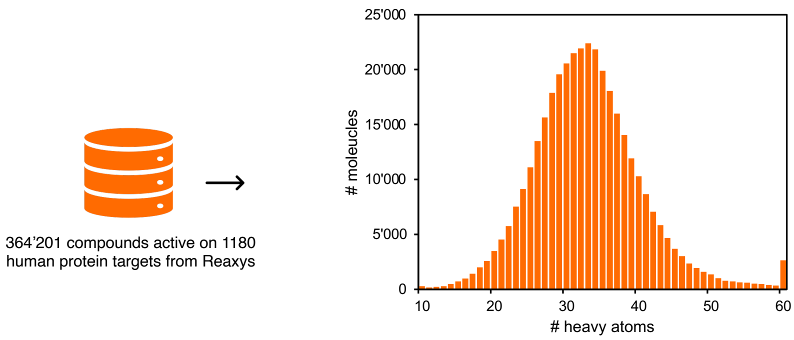

Supplementary Fig. 6: **Test set split into heavy atom classes**. The histogram gives the number of molecules in the test set of a function of the number of heavy atoms.

Supplementary Table 1: 10-fold cross-validation of the logistic model.

# Heavy atoms	MCC_{CV}	Standard Deviation	Precision (PPV)	Recall (TPR)
≤10	0.655	0.084	0.794	0.575
11	0.672	0.075	0.789	0.607
12	0.748	0.038	0.813	0.743
13	0.787	0.024	0.841	0.771
14	0.776	0.045	0.840	0.754
15	0.805	0.030	0.851	0.786
16	0.829	0.030	0.881	0.801
17	0.829	0.024	0.872	0.814
18	0.850	0.018	0.884	0.844
19	0.856	0.018	0.884	0.849
20	0.866	0.016	0.900	0.859
21	0.874	0.018	0.905	0.864
22	0.873	0.019	0.901	0.868
23	0.877	0.010	0.907	0.871
24	0.892	0.014	0.916	0.888
25	0.891	0.009	0.921	0.883
26	0.908	0.010	0.929	0.902
27	0.914	0.007	0.936	0.908
28	0.911	0.008	0.930	0.909
29	0.921	0.008	0.939	0.918
30	0.927	0.010	0.944	0.923
31	0.932	0.008	0.946	0.930
32	0.937	0.009	0.948	0.938
33	0.934	0.008	0.949	0.933
34	0.926	0.009	0.944	0.923
35	0.939	0.009	0.953	0.936
36	0.937	0.009	0.949	0.937
37	0.933	0.008	0.949	0.931
38	0.931	0.009	0.944	0.931
39	0.936	0.008	0.952	0.933
40	0.929	0.011	0.947	0.923
41	0.934	0.011	0.939	0.943
42	0.919	0.015	0.942	0.913
43	0.932	0.010	0.944	0.935
44	0.934	0.015	0.941	0.941
45	0.937	0.009	0.949	0.939
46	0.909	0.023	0.937	0.904
47	0.923	0.020	0.929	0.935
48	0.917	0.019	0.917	0.929
49	0.926	0.021	0.937	0.937
50	0.918	0.020	0.936	0.920
51	0.906	0.020	0.908	0.922
52	0.924	0.025	0.944	0.922
53	0.923	0.016	0.940	0.936
54	0.893	0.024	0.906	0.902
55	0.891	0.021	0.922	0.881
56	0.901	0.025	0.925	0.901
57	0.896	0.033	0.927	0.904
58	0.885	0.028	0.913	0.897
59	0.938	0.019	0.947	0.934
≥60	0.915	0.038	0.919	0.931

Supplementary Table 2: example of the raw training data as extracted from ChEMBL.

Bioactivity[a]	Standardized SMILES	Compound CHEMBLID	#Heavy atoms	Activity type	Activity value	Activity unit	Assay type	Confidence score	Target type	Target CHEMBLID	Organism	UniProtID
A	CN1CCCCC1C1=C(NC2=CC=CC=C12)C1=CC=CC=C1	CHEMBL43697	22	Ki	2000.000	nM	B	8	SINGLE PROTEIN	CHEMBL225	Homo sapiens	P28335
I	CCC(=O)N[C@H]1C=C(O[C@@H]([C@H](O)[C@H](O)CO)[C@@H]1NC(C)=O)C(O)=O	CHEMBL4084317	24	IC50	500000.000	nM	B	9	SINGLE PROTEIN	CHEMBL4174	Homo sapiens	Q8WWR8
G	OC(=O)C1=CC=C(C=C1)N1N=CC2=CC=CC=C2C1=O	CHEMBL3337973	20	IC50	50000.000	nM	B	9	SINGLE PROTEIN	CHEMBL4342	Homo sapiens	O15496

[a]A: active datapoints (≤ 10μM); I: inactive datapoints (≥ 100μM); G: grey area
